# A Study on the Fire-Retardant and Sound-Proofing Properties of Stainless Steel EAF Oxidizing Slag Applied to the Cement Panel

**DOI:** 10.3390/ma16083103

**Published:** 2023-04-14

**Authors:** Chuan-Wen Chou, Hung-Ming Lin, Guan-Bang Chen, Fang-Hsien Wu, Chen-Yu Chen

**Affiliations:** 1 Department of Architecture, National Cheng Kung University, Tainan City 701, Taiwan; 2 Acoustics Laboratory, MingDao University, Changhua City 523, Taiwan; 3 Sustainable Environment Research Laboratory (SERL), National Cheng Kung University, Tainan City 701, Taiwan; 4 Department of Aeronautics and Astronautics, National Cheng Kung University, Tainan City 701, Taiwan; 5 Research Center for Energy Technology and Strategy (RCETS), National Cheng Kung University, Tainan City 701, Taiwan

**Keywords:** circular economy, EAF oxidizing slags, high-value building materials, fire retardancy, sound-proofing

## Abstract

Because of incomplete recycling resource management and technology development, inorganic sludge and slag has been misused in Taiwan. The recycling of inorganic sludge and slag is a pressing crisis. Resource materials with a sustainable use value are misplaced and have a significant impact on society and the environment, which greatly reduces industrial competitiveness. To solve the dilemma of EAF oxidizing slag recycled from the steel-making process, it is important to find solutions to improve the stability of EAF oxidizing slags based on the innovative thinking of the circular economy. We can improve the value of recycling resources and solve the contradiction between economic development and environmental impact. The project team intends to investigate the development and application of reclaiming EAF oxidizing slags blended with fire-retardant materials, which will integrate R&D work from four different aspects. First, a verification mechanism is carried out to establish stainless steel furnace materials. Suppliers must be assisted in conducting quality management for EAF oxidizing slags to ensure the quality of the materials provided. Next, high-value building materials must be developed using slag stabilization technology, and fire-retardant tests must be conducted on the recycled building materials. A comprehensive evaluation and verification of the recycled building materials must be undertaken, and high-value green building materials must be produced with fire-retardant and sound-proofing characteristics. Integration with national standards and regulations can drive the market integration of high-value building materials and the industrial chain. On the other hand, the applicability of existing regulations to facilitate the legal use of EAF oxidizing slags will be explored.

## 1. Introduction

The circular economy describes an economic system that is based on business models which replace the ‘end−of−life’ concept with reducing, alternatively reusing, recycling, and recovering materials in production/distribution and consumption processes, but even the European Commission listed the three major failures from market, regulatory, and information [1,2].

- Market failures: The present market mechanism cannot provide a reliable procedure, resulting in an inability to achieve a market consensus on the quantification of environmental impact. This results in insufficient comparability of the results of environmental impact assessments. The further unfairness of competition appears in the form of environmental performance and company cost.
- Regulatory failure: The environmental footprint methodology of products and organizations is based on a voluntary basis only. Although these methods have specifically established the methodological basis in the Recommendation 2013/179/EU12, due to the unlimited use of alternative methods, their environmental assessment results cannot be compared and thus cannot be effectively supervised through acceptable regulations.
- Imperfect information: Product environment-related performance can only be provided to market participants in specific categories of specific products. If it is required to be included in the ‘best−in−class’ product category from the EU Ecolabel, the environmental performance of the product is derived from such specific products and is also included in the Ecolabel, and equivalent national schemes (e.g., Nordic Swan, Blue Angel, etc.) have the same situation. This complicates the presentation of the product’s environmental performance and generates information delays, which in turn can affect the reliability of the information.

Recycling is a major issue in Taiwan today, as the steelmaking process in electric arc furnaces (EAFs) generates up to 15% of slag per ton of steel. There were also nearly 150 megatons of EAF slag produced per year in Taiwan. Although the EPA and other related agencies proposed many regulatory policies, there are over 200 steel industrial companies with application manuals used for dealing with these industrial wastes, but only a few waste management companies, not to mention fewer application products besides cement, brick, or road and civil construction. Moreover, the use of EAF slags is forbidden in the building of structural parts [T1]. Materials added to the steel melt directly before the end of the reheating process are not fully embedded in the slag structure, so they can be found in the slag as free oxides (CaO and MgO). Compared to blast furnace slag, the stainless EAF oxidizing slag shows a considerably higher CaO/SiO_2_ ratio, has more weight, has a higher hardness and density, is less porous, and is highly resistant to polishing and wear, and as such is very suitable for road building or the improved mechanical strength of hardened concretes. However, it may be subjected to volumetric instability problems, due to the possible expansion of free CaO and free MgO. The common method, announced by EPA in 2019, proposed that the slags were stabilized by continued pressurized steam over 3 h under 21 kgf/cm^2^, before which the EAF slags were used as aggregates on construction sites. Given that too many types of incineration slags are already being using in road engineering and sewage construction in Taiwan, the stainless steel EAF oxidizing slag may not be able to be fully used in these applications, as it lacks diversity [3].

An overview of the current diversification of building materials: The decoration materials market has grown in recent years, and the raw material requirement of heat- and sound-proof fire retardants is urgent, particularly after COVID-19 has slowed down the import business, raw materials has been recycled from industrial wastes, and new methods have been developed as new solutions for the raw material shortage. The EPA has no definite regulatory policies on EAF slag application on decoration materials; even when compared to regular decoration materials, EAF slags could increase the fire retardancy, reduce heat transmission by a factor of two, and increase sound insulation by a factor of 1.5 [T2]. Unfortunately, public impressions of recycled products were negative, because most products were downcycled, inefficient, and harmful to health. Consumers and businesses need reliable, comparable, and verifiable information to make sustainable decisions.

This study will focus on stabilizing the stainless EAF oxidizing slag without high pressure steam, and recycling the stainless EAF oxidizing slag with fly−ash could generate a geopolymer precursor, which suggests that the bonding glue on decoration material manufacturing could replace cement. Based on demands of building materials, it is economical, thermally stable, sound-proof, easily workable, eco-friendly, cement-free, and durable, with low energy consumption. Apart from geopolymers, a standard decoration material still needs a certificate of safety, health, and physical performance; therefore, the heat release rate test will evaluate the fire retardancy and thermal insulation rates, while the impedance tube will estimate the sound insulation performance. The toxicity characteristic leaching procedure (TCLP) will determine the mobility of both organic and inorganic analytes present in the liquid and solid of the finale formation. Looking forward to lower CO_2_ emissions, the optimum utilization of natural resources and the utilization of waste materials is more cost-effective in long-life infrastructure construction, provides social and financial benefits, and generates employment opportunities.

## 2. Materials and Methods

### 2.1. Slag Modification and Inorganic Geopolymer Technology

**Figure 1:**
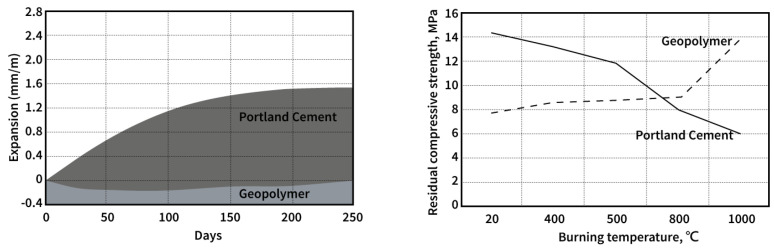
By comparing the alkali aggregate reaction (ASTM C227) between the geopolymer and Portland cement shown after 90 days, the geopolymer was found to have no alkali reaction with long−term durable performance. Even the test sample was under over 1000 °C and the residual compressive strength still retained approximately 14 MPa, proving that the geopolymer combination had better resistance and durable performance [7].

The geopolymer was introduced by the Davidovits in 1978 and is made by the alkaline solution’s activation (consists of sodium or potassium silicate and sodium or potassium hydroxide) of the high alumina silica-rich materials. Ceramic composites can establish a bond between alumina and silica. Geopolymer-based concrete using fly–ash can allow the construction industry to replace Portland-cement-based concrete with comparable structural properties [4]. Geopolymers, such as natural zeolite minerals, represent a class of three-dimensionally networked aluminosilicate materials. Due to its superior mechanical and physical properties, such as non-combustible, heat-resistant, fire/acid-resistant, sound-proof, and easy-to-reach features, and given the fact that they can be formed at low temperatures, as shown in Figure 1, geopolymers have been gradually attracting worldwide attention as potentially revolutionary green materials [5,6].

Pressurized steam can stabilize free CaO and free MgO into Ca(OH)_2_ and Mg(OH)_2_ under 21 kgf/cm^2^ pressure over 3 h, and the Ca(OH)_2_ and Mg(OH)_2_ will slow the reaction of cement hydration. These contents will even increase strength, but easily fall off from the concrete structure, increasing the amount of steam equipment expenditure and creating more CO_2_ emissions. Therefore, reducing the scale of EAF oxidizing slags to 0.42 mm (40 mesh) will decrease the possible expansion volume and add alumina silica-rich materials (such as fly ash.) Inorganic sludge containing SiO_2_ and Al_2_O_3_ will also generate more meticulous calcium silicate hydrate (C−S−H) gel. When these C−S−H gels fill the gap of concrete structures, the mechanical strength will be enhanced after conservation and the micropore in the material structure will reduce the heat transmission and stabilize possible expansion [8,9].

This study will apply fly–ash-based geopolymers as binders from the f−CaO of slags to stabilize EAF oxidizing slags [10,11]. To establish the possibility of using it for decoration materials, the characterization and chemical composition of the fly−ash and EAF oxidizing slag would need to be analyzed by inductively coupled plasma mass spectrometry (ICP−MS) [12,13,14]. The mechanical performance would need to be examined based on compressive strength, length change, and autoclave expansion, and the optimized combination would need to be examined by TCLP in reference to NIEA R201.14C. Details of the experiments are listed in Table 1 and the experiment combinations are listed in Table 2.

### 2.2. Visual Fire-Retardant Technology

Fire retardants may be the necessary property of building materials. For high-value applications of circular materials, the heat performance and fire-retardant requires the proportion of materials to be verified in the testing step to achieve building application. The related standards are listed below in Table 3. After making designs according to the engineering application characteristics of the relevant national standards of various cement panels, the fire-retardant test of all levels is carried out according to the combustion test method of building interior decoration materials. The study intends to conduct relevant fire-retardant experiments with a self-designed and built combustion detection system.

**Figure 2:**
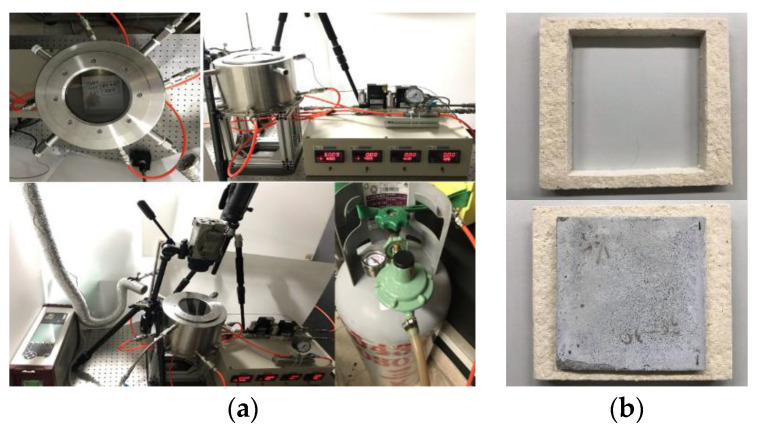
Miniaturized fire-retardant material test furnace system and specimens fixing frame. (**a**) System assembling. (**b**) The fixing patterns of specimens.

Generally, the size of the test material for fire-retardant material testing requires a larger size (length × width = 20 cm × 20 cm). According to that, a miniaturized fire-retardant material test furnace system is built, as shown in Figure 2. This test furnace is made of stainless steel 316. The infrared ceramic furnace is used as the heat source at the bottom. The cubes (with sizes of 10 cm and 5 cm) and the plate (with a thickness of 2 cm) are placed on the top. Once air inlet provides air, the upper four air outlets can be used for exhaust gas collection and analysis at the same time. A high-temperature quartz plate is placed on the upper cover of the furnace body as an observation window, which can be used to measure and record the whole process of the test piece and measure the temperature with an infrared thermal imager as a test platform. In the specimen part, the ceramic fiber board can be cropped into a suitable size to close-fit the specimen. This test refers to CNS 6532, CNS 14705 fire-retardant test specifications for decoration materials and building materials. The flame-retardant materials must be continuously heated for 5, 10, and 20 min to be defined as fire-retardant grades 3, 2, and 1. In addition, it can also be provided if long-term heating is required. Manufacturers with test requirements are used as test platforms.

### 2.3. Sound Transmission Loss Measurement in Small Specimens

Some provisions of the Architectural Design and Construction Code and the Architectural Structure Code were revised in the Architectural Technical Regulations in 2016. In the sound-proofing part of the Architectural Design and Construction Code, the sound insulation performance of partition walls was added as the evaluation standard for acoustic-proof structures, which is evident in the current urban architectural space. Due to the significant importance of the sound insulation performance of building materials, under the current domestic industry and policy development direction, seeking sound insulation building materials that consider both circular economy and high performance is the current focus of application development.

**Figure 3:**
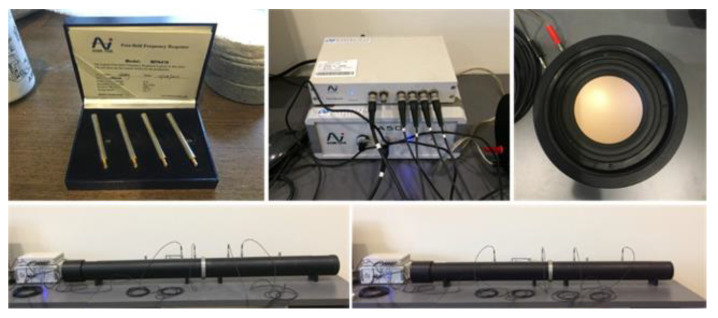
Impedance tube system and 1/4″ microphone.

The sound insulation test is demanded on a full scale and a complete construction of building materials is carried out. However, it is difficult to achieve testing specifications in the early development stage of materials. In order to verify sound-proofing properties in relation to mechanical properties and heat performance in time, which are used for adjusting material proportions in agility, the study applies an impedance cube system to determine sound transmission loss and evaluate the sound-proofing properties of specimens in different material proportions. The system includes a cube with diameter of 10 cm, a sound source of 97–100 dB is emitted by the loudspeaker, and the values at both ends are measured with a 1/4″ microphone and then analyzed with a four-channel analyzer to obtain the transmission loss value measurement of the normal incident sound energy. The system is shown in Figure 3. The measurement specification is adopted as ASTM E1050 (ISO 10534) with a range of 80~1600 Hz.

**Figure 4:**
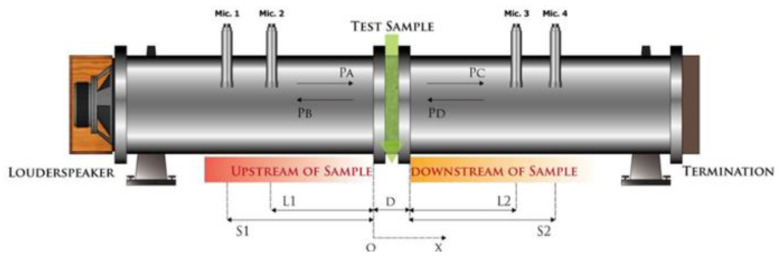
Content of sound transmission loss measurement.

The test body prepared by the above is placed in the center of the tube wall, and the gap around the test body is filled to avoid sound leakage affecting the measurement value. The test is divided into two test procedures of 80~500 Hz and 400~1600 Hz. The distance between S1 and S2 is adjusted to measure the different frequency bands. Finally, the numerical value is synthesized to obtain the transmission loss performance of the overall frequency band of 80~1600 Hz. The content of system is shown in Figure 4.

The test materials for the sound transmission loss test are made from the raw materials, and the poured test materials with a thickness of 20 mm are used as the test samples for the transmission loss test. EAF oxidizing slags are used as raw materials. After EAF oxidizing slags are crushed, screened, and mixed, the round mold is poured through the modification procedure, and the preparation is completed after 28 days of curing. Its high specific gravity can correspond to the subsequent development of the heavy cladding panel; the control part is made of natural cement with the same proportion as the sample.

## 3. Results

In light of the results used for evaluating EAF oxidizing slags as building materials, the basic mechanic characteristics showed a higher strength than the same types of cement panels. Based on environmental compatibility, the results of the toxicity characteristic leaching procedure can also be applied to building materials. In regard to fire-retardant and sound-proofing properties, the results also showed 1.5 to 2 times higher performance values compared to the normal cement panel. There was also an advanced description for several parts below.

### 3.1. Application of Inorganic Polymerization Technology to Controllable Density Materials

**Figure 5:**
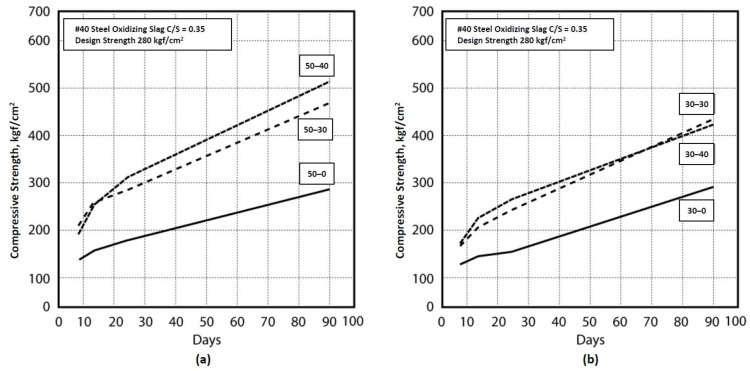
Compressive combination strengths on the 0.35 ratio. (**a**) EAF oxidizing slag adjunction with a percentage of 50%. (**b**) EAF oxidizing slag adjunction with a percentage of 30%.

**Figure 6:**
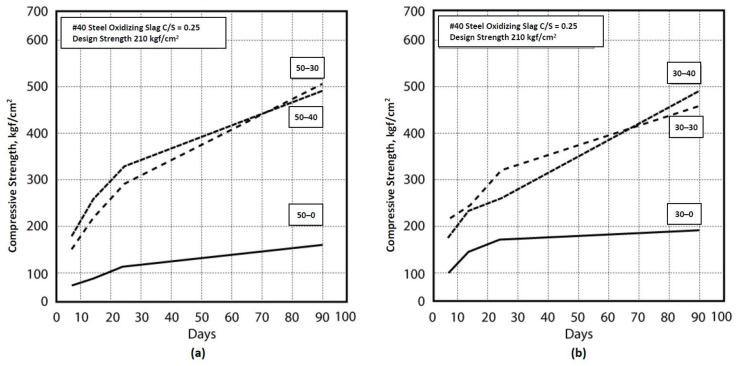
Compressive combination strengths on the 0.25 ratio. (**a**) EAF oxidizing slag adjunction with a percentage of 50%. (**b**) EAF oxidizing slag adjunction with a percentage of 30%.

These combinations were tested at curing times of 7, 14, 28, and 90 days with average values recorded, and these strength trends are shown in Table 4 and Figure 5 and Figure 6. The 28th-day strength of the 0.35 cement−sand ratio was 308.76 kgf/cm^2^, showing a combination of 50 % EAF oxidizing slag and 40 % fly−ash, with a strength of 527.8 kgf/cm^2^ on the 90th day. The 28th-day strength was 259.76 kgf/cm^2^ when the EAF oxidizing slag was 30% and the fly−ash 40%, and the long-term strength was 419.78 kgf/cm^2^ on the 90th day. Among these combinations, the strength development was sluggish after 90 days when the cement−sand ratio was 0.35. The cement−sand ratio was 0.25, and the strength was 255.96 kgf/cm^2^ on the 28th day (with an EAF oxidizing slag of 30% and a fly−ash of 40%) and 501.29 kgf/cm^2^ on the 90th day (with an EAF oxidizing slag of 50% and a fly−ash of 30%). The highest compressive strength was 1.86 times greater than the general concrete from 28 to 90 days when the combined cement−sand ratio 0.35, with an EAF oxidizing slag of 30% and fly−ash of 30%. The combination was 1.91 times than general concretes, when cement−sand ratio was 0.25, with an EAF oxidizing slag of 30% and a fly−ash of 40%.

The waterproof index is an essential consideration for decoration materials [15,16], and lower absorption coefficients indicate better performance in terms of durability, stability, and construction purposes [17,18], and the water absorption results of each combination are shown in Table 5. The water absorption ratio was 15~18%, and the specific gravity range was 1.70~1.85 when the cement−sand ratio was 0.35. The water absorption ratio was 11~13%, and the specific gravity range was 1.90~2.00 when the cement−sand ratio was 0.25. The water absorption ratio was 12~21%, and the specific gravity range was 1.65~1.88 when the cement−sand ratio was 0.15. Through the water absorption results of different combinations, the increased fly−ash addressed the gap of concrete structure which reduced the absorption ratio, and vice versa.

**Figure 7:**
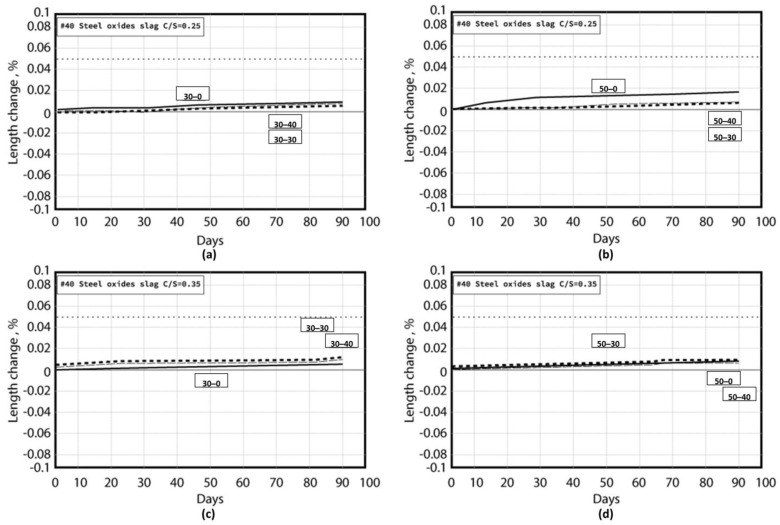
Length changes in different combinations. (**a**) EAF oxidizing slag adjunction with a percentage of 30% and a C/S ratio of 0.25. (**b**) EAF oxidizing slag adjunction with a percentage of 50% and a C/S ratio of 0.25. (**c**) EAF oxidizing slag adjunction with a percentage of 30% and a C/S ratio of 0.35. (**d**) EAF oxidizing slag adjunction with a percentage of 50% and a C/S ratio of 0.35.

To evaluate the volume stability of combinations, the experiment was based on the CNS 14603 (“Use of apparatus for the determination of length change of hardened cement paste, mortar, concrete”) [19,20], but there were no expansion standard values; therefore, the expansion standard value reference from ASTM C33 was measured when the expansion value was 0.05% at 90 days. Figure 7 shows that all combination lengths had changing results under 0.035%, regardless of the cement−sand ratios of 0.25 and 0.35. The expansion results are shown in Table 6. The particle size of the EAF oxidizing slag was found to affect the stability, with smaller particles emerging less damaged or burst.

### 3.2. Environmental Compatibility Verification

The environmental compatibility of circular building materials in this study was evaluated in light of raw materials and concrete building materials through the toxicity characteristic leaching procedure (TCLP). The EAF oxidizing slag in this study was oxidizing slag with sizes in sand grading. After being crushed and passed through a No. 200 sieve, the specimen of raw materials was subject to element separation by ICP−OES, and results are shown in Table 7 and Table 8. The TCLP results of concrete building materials with EAF oxidizing slag are shown in Table 9, and it can be found that the dissolution results of the proportions of each group are lower than the standard dissolution values of hazardous industrial wastes, which are in line with the current national standards.

The oxidizing slag is mainly composed of calcium, silicon, aluminum, magnesium, etc. The secondary components are heavy metal components, of which the heavy metal chromium content is the highest. The results of the upstream percolation test method show that EAF oxidizing slags provided an alkaline substance with only trace amounts of dissolved heavy metals (such as barium (Ba), chromium (Cr), and lead (Pb)) [21]. The EAF oxidizing slag and its concrete samples with different pH values showed that the EAF oxidizing slag dissolved many kinds of heavy metals, and the dissolved amount was high, while the heavy metals dissolved in the concrete samples were mostly dissolved in trace amounts or below the limit of quantification. Concrete samples are non-corrosive, and the dissolution behavior of heavy metals was measured in the bulk diffusion test, which showed that the heavy metals in them were not easily released in the environment, which was in line with environmental safety [22]. The amount of soluble inorganic components was determined. It was estimated that the number of heavy metals in concrete specimens containing EAF oxidizing slags can be fixed and stabilized in concrete specimens after being transformed into building materials.

### 3.3. Fire-Retardant Properties of EAF Oxidizing Slags Used in Building Materials

**Figure 8:**
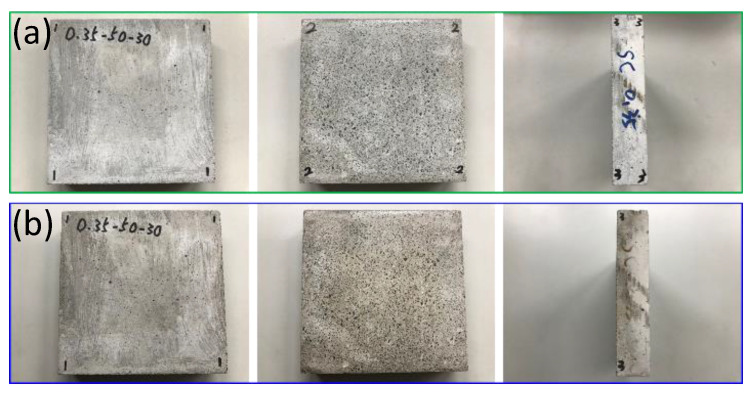
#40−50−30−C/S = 0.35. (**a**) Before testing. (**b**) After testing.

**Figure 9:**
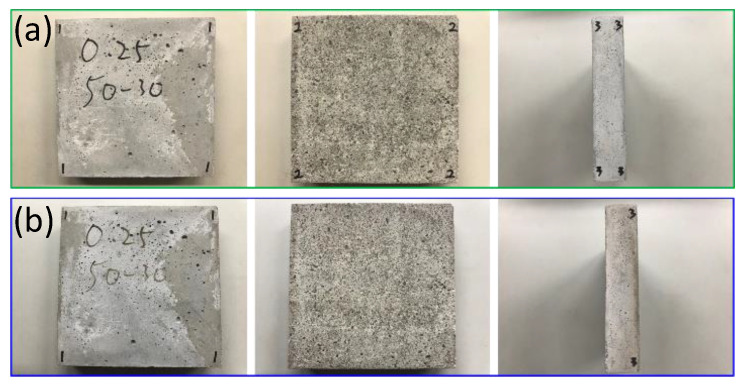
#40−50−30−C/S = 0.25. (**a**) Before testing. (**b**) After testing.

**Figure 10:**
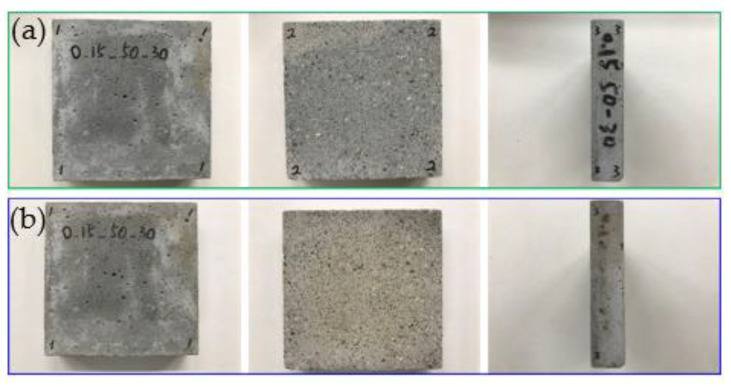
#40−50−30−C/S= 0.15. (**a**) Before testing. (**b**) After testing.

The team used a self-built small test furnace, coupled with thermal image analysis, to study the thermal characteristics of EAF oxidizing slag test pieces. The test piece used an infrared thermal imager and a high-quality camera to monitor the temperature change and appearance of the test piece. For the EAF oxidizing slag series specimens, in the series with a soil−cement ratio of 0.35, the EAF oxidizing slags refractory admixture specimens with different proportions were placed at room temperature for thermal testing for cooling. The heating surface in contact with the flame had a large gray black area, with no small cracks on the surface, while the back of the heating surface had no burns, penetrations, cracks, and holes, and the only part of the surface was gray black. After the thermal test, the surrounding wall of the test piece appeared coffee black because the ceramic fiber board used to fix the test piece turned coffee black after being heated and stained on the test piece. This was not caused by the properties of the specimen itself. A comparison chart of the EAF oxidizing slags and refractory admixture with different ratios of 0.35, 0.25, and 0.15 was also found (as shown in Figure 8, Figure 9 and Figure 10); after heating, the results were almost like the heating results of the specimen with a soil−cement ratio of 0.35.

**Figure 11:**
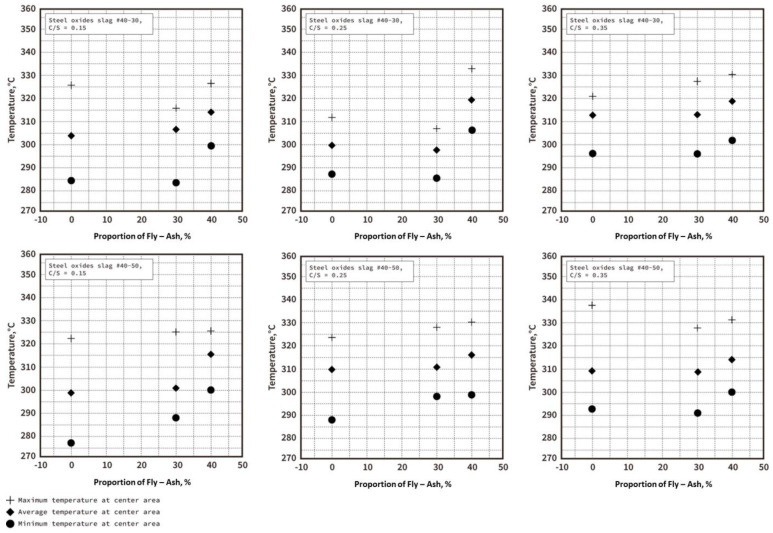
Temperature distribution of the EAF oxidizing slag specimens in three ratio settings.

**Figure 12:**
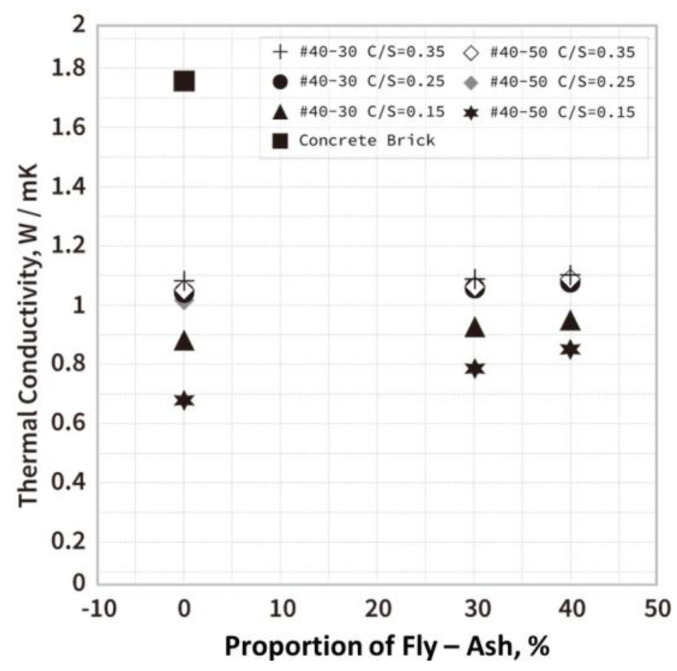
Thermal conductivity of EAF oxidizing slag specimens in three ratio settings.

**Figure 13:**
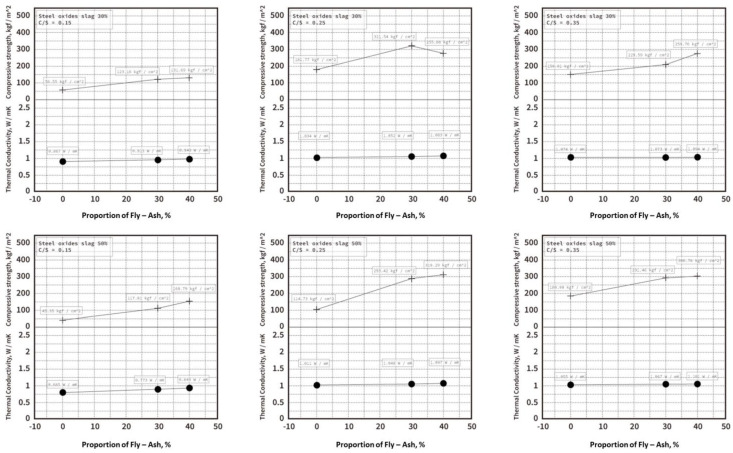
Compressive strength and thermal conductivity of EAF oxidizing slag specimens with different ash replacements.

Under the three soil−cement ratio designs (C/S = 0.35, C/S = 0.25, and C/S = 0.15), the test pieces with different ratios of EAF oxidizing slags flame-retardant admixtures were subjected to the thermal test results (as shown in Figure 11), showing that the overall temperature range of the temperature distribution of the 0.35 soil−cement ratio was close and higher, while the temperature distribution of the 0.15 soil−cement ratio series was very different and the low temperature was lower. It can also be found that, overall, when fly–ash addition helped to modify the properties of the EAF oxidizing slag, it increased the temperature uniformity when heated. A similar trend was also found in the thermal conductivity (k) results of the test materials, as shown in Figure 12, where the thermal conductivity of the soil−cement ratios of 0.35 and 0.25 ranged between 1 and 1.1, so the temperature was distributed after heating. The thermal conductivity of the soil−cement ratio of 0.15 ranged from 0.6 to 0.9, so the temperature distribution after heating was relatively low. However, the overall series still maintained good heat resistance and thermal insulation effects and was superior to the commercial heavy concrete bricks (thermal conductivity (k) of 1.8 W/mK) [23,24]. In addition, we also compared the initial compressive strength and thermal conductivity of the three soil−cement ratio designs (as shown in Figure 13). The strength still showed an increasing trend, and the thermal conductivity was not affected by the addition of fly–ash, and its numerical value was not very different.

In this study, according to the experimental results on the thermal characteristics of the EAF oxidizing slag test piece, the test piece conditions with excellent compressive strength values and improved thermal insulation and thermal conductivity coefficients were selected (for example: #40−30−40−C/S = 0.35, #40−50−40−C/S = 0.35, #40−30−30−C/S = 0.25, #40−30−40−C/S = 0.25, #40−50−30−C/S = 0.25, #40−50−40−C/S = 0.25) for cone calorimeter analysis, and the “Test method for combustion heat release rate of building materials—Part 1: Cone calorimeter method” (CNS 14705−1), which is a standard test method that is used to evaluate fire-retardant grades of building materials. Furthermore, the soil−cement ratio C/S = 0.35 and the soil−cement ratio C/S = 0.25 developed in this study were tested by a cone-measuring instrument, and it was found that the heating lasted 1200 s. Over time, the total heat release of each test piece was far smaller than 8 MJ/m^2^, which means that the fire retardancy of each test piece was verified under fire retardancy level 1 [25], which is helpful for the fire retardancy of building materials across a wider application range.

### 3.4. Soound-proof Properties of EAF Oxidizing Slags Used in Building Materials

**Figure 14:**
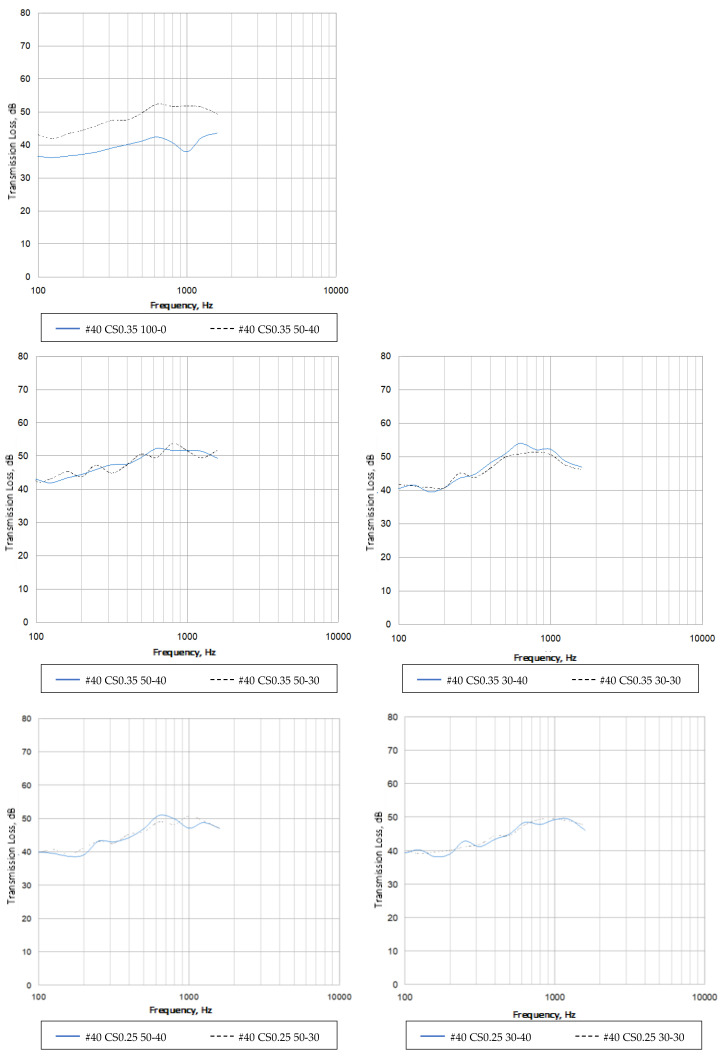
Sound transmission loss curves of different combinations.

According to the transmission loss of each sample, as shown in Table 10 and Figure 14, the construction method mainly led to stiffness and strength, which is reflected in the effect of specific gravity and thickness on the transmission loss performance. Based on most of the application scenes of the panel used in this study, it was used for the main body of the surface material, so 80~1600 Hz was used as the comparison range of transmission loss performance [26,27]. Considering the stabilization needs of the EAF oxidizing slag, the test was carried out with the optimal proportion; the proportion of fly–ash can be adjusted to confirm its benefits. The following points will analyze the transmission loss characteristics of various materials, and then confirm the benefits of different application methods through a comprehensive comparison of each material.

EAF oxidizing slags were used as the main admixture to cement panels, and the control group was a test material of the same thickness panel with 100% natural sand. After this ratio optimization, the original low-frequency disturbance was effectively eliminated. Its overall performance reached more than 40 dB, and it then exceeded 50 dB above 500 Hz. It can be confirmed that it has significant high-transmission-loss characteristics. The follow-up application direction can also develop heavy sound insulation materials.

The effect of the fly–ash mixing ratio on sound transmission loss was further compared under different cement–sand ratio conditions. The low frequency of the 0.35 ratio was 3–5 dB higher than that of the 0.25 ratio, the middle- and high-frequency parts were similar, and some frequency bands were 1–3 dB higher. It can be confirmed that the improvement in the strength of the EAF oxidizing slag admixture was reflected in the stiffness control of the middle and low frequencies, thereby improving the sound insulation value; the performance of the middle and high frequencies slightly improved due to the increase in density mass, but this was not significant.

Based on the above three samples and the control group, which is a natural cement slab sample, the transmission loss performances were compared to confirm the feasibility of using EAF oxidizing slags as cement panels with sound insulation. The cement panel prepared from the oxidizing slag can be used as a heavy-duty building decoration material for subsequent applications. The performance of each frequency band was significantly higher than that of the control group, with a difference of 8–10 dB for each frequency. Furthermore, the transmission loss reduction in part of frequencies with lower or unstable testing results may be related to the consistency of heterogeneous materials and impacts on stiffness and strength.

## 4. Discussion

In this study, the EAF oxidizing slags below the 40 mesh (below 0.42 mm) were effectively used to reprocess the cement products, and after appropriate modification with fly–ash using the low-energy-consuming silicon-based modification method, the issue of expanding EAF oxidizing slag products was successfully solved. Therefore, its appearance and expansion rate were lower than 0.09%.

The results of the compressive strength test showed two optimize proportions in the series of the C/S ratio of 0.25. One suggests that the EAF oxidizing slag replaced 30% sand and ash replaced 30% EAF oxidizing slag, and the compressive strength at the 28th day age was 321.54 kgf/cm^2^. The other suggests the EAF oxidizing slag replaced 50% sand and 40% EAF oxidizing slag was replaced by ash, and the compressive strength at the 28th day was 308.8 kgf/cm^2^, and the compressive strength increased to 495.5 kgf/cm^2^ on the 365th day. With a C/S ratio of 0.35, the compressive strength was better when the EAF oxidizing slag replaced 50% sand and ash replaced 40% EAF oxidizing slag, and the compressive strength at the 28th day was 308.8 kgf/cm^2^, and the compressive strength increased to 495.5 kgf/cm^2^ on the 365th day.

Both the 0.25 series and 0.35 series C/S ratios showed that the compressive strength was twice the design strength, so the use of EAF oxidizing slags could effectively reduce the amount of cement and improve the compressive strength of the specimen. It was found that the thermal insulation performance of the modified the EAF oxidizing slag cement product was better than that of the commercially available heavy concrete panel, with nearly double the thermal insulation performance advantage. In addition, the sound transmission loss test results of the building material were relatively better than the existing heavy concrete panel, with the advantage of improving the sound insulation performance by about 40%.

The results of the TCLP test showed that the heavy metal chromium (Cr) and heavy metal lead (Pb) of the oxidizing slag were both lower than 0.1 ppm in the TCLP test results, which is in line with the standard specification value limit [28] of heavy metal barium (Ba). The leaching value ranged between 0.3 ppm and 0.7 ppm, which indicates trace leaching, but far below the standard specification value of 100 ppm. Other heavy metals such as arsenic (As), cadmium (Cd), hexavalent chromium (Cr^6+^), copper (Cu), selenium (Se), mercury (Hg), etc., were all below the quantitative limit, which is in line with environmental safety regulations [29].

## 5. Conclusions

According to results of different proportions of EAF oxidizing slags and fly–ash, for optimizing purposes, a cement and sand ratio of 0.35, an EAF adjunction percentage of 50%, and a fly–ash adjunction percentage of 40% are required. Panels in this proportion provide 1.5 to even 2 times greater strength, fire retardancy, and sound-proofing performance than the general ones. Combined with its circular materials and low-energy-consumption modification process, there is no doubt about environmental leaching and compatibility in current specifications, and has nearly doubled the late strength, nearly doubled the thermal insulation, and improved sound-proofing properties, both demonstrating fire-retardant properties. Therefore, through the results of this research, it can be confirmed that the EAF oxidizing slags have characteristics associated with the subsequent development of fire-retardant and sound-proofing building components.

## 6. Patents

This research was partially supported by the Ministry of Science and Technology of Republic of China under the grant number MOST 107−2218−E−006−057. The EAF oxidizing slags were totally supported by Walsin Lihwa Corporation.

## Figures and Tables

**Table 1:** Mechanical performance examination.

Examine Subjects	Details and Standards
Mechanical and physical properties	Specific density, particle size distribution, XRF
Combination	1. C/S * = 0.35, 0.25 2. Substitute slag percentage 30%, 50% 3. Fly−ash percentage 0%, 30%, 40%
Method to test the compressive strength of cylindrical concrete specimens	CNS 1232
Method to test the apparent porosity, water absorption, and specific gravity of refractory bricks	CNS 619
The use of apparatus for the determination of length changes in hardened cement paste, mortar, and concrete	CNS 14603
Method to test for the autoclave expansion of Portland vement	CNS 1258
Toxicity characteristic leaching procedure	NIEA R201.14C

* C/S indicates the cement−sand ratio.

**Table 2:** EAF oxidizing slag decoration material combination.

Design Strength (kgf/cm^2^)	Cement−Sand Ratio (C/S)	EAF Oxidizing Slags Adjunction Percentage *	Fly–Ash Adjunction Percentage **	Proportion Code
280	0.35	30% 50%	0%	CS0.35 30−0
			30%	CS0.35 30−30
			40%	CS0.35 30−40
210	0.25	30% 50%	0%	CS0.25 30−0
			30%	CS0.25 30−30
			40%	CS0.25 30−40

* The adjunction indicates the percentage of sand replaced by EAF oxidizing slags. ** The adjunction indicates the percentage of EAF oxidizing slags replaced by the fly–ash.

**Table 3:** Related standards of building material properties.

Demands of the Basic Properties of Board		Fire-Retardant Verification	
Fiber-reinforced cement sidings	CNS 11699	Method to test for the incombustibility of the interior finish materials of buildings	CNS 6532
Fiber-reinforced cement boards	CNS 13777		
Regenerated fiber cement boards	CNS 14890	Method to test for the heat release rate for building materials (part 1: cone calorimeter method)	CNS 14705−1
Fiber cement boards	CNS 3802		

**Table 4:** The compressive strength values of EAF oxidizing slag bricks.

Combination	Average Compressive Strength (kgf/cm^2^)			
	At Day 7	At Day 14	At Day 28	At Day 90
#40 CS0.35 30−0	124.74	145.04	150.80	296.25
#40 CS0.35 30−30	175.19	204.50	229.25	425.51
#40 CS0.35 30−40	179.44	220.97	259.76	419.78
#40 CS0.35 50−0	145.41	165.11	178.43	298.41
#40 CS0.35 50−30	208.35	251.31	291.55	474.61
#40 CS0.35 50−40	195.83	251.61	308.76	527.80
#40 CS0.25 30−0	100.03	149.57	170.20	190.61
#40 CS0.25 30−30	217.62	247.69	321.54	455.42
#40 CS0.25 30−40	179.68	230.28	255.96	489.80
#40 CS0.25 50−0	58.35	93.18	110.24	157.91
#40 CS0.25 50−30	151.89	215.23	293.12	501.29
#40 CS0.25 50−40	180.72	257.36	323.42	497.54

**Table 5:** Water absorption of EAF oxidizing slag bricks.

Combination	Apparent Specific Gravity (Gs, ssd)	Water Absorption (%)	Combination	Apparent Specific Gravity (Gs, ssd)	Water Absorption (%)
CS0.35 30−0	1.72	17.20	CS0.25 50−0	1.71	17.52
CS0.35 30−30	1.84	16.31	CS0.25 50−30	1.92	12.24
CS0.35 30−40	1.84	16.42	CS0.25 50−40	1.94	11.04
CS0.35 50−0	1.75	17.96	CS0.15 30−0	1.74	18.02
CS0.35 50−30	1.84	15.63	CS0.15 30−30	1.88	13.24
CS0.35 50−40	1.83	15.30	CS0.15 30−40	1.86	13.46
CS0.25 30−0	1.83	14.17	CS0.15 50−0	1.65	21.46
CS0.25 30−30	1.95	12.88	CS0.15 50−30	1.82	14.14
CS0.25 30−40	1.96	12.04	CS0.15 50−40	1.83	12.53

**Table 6:** Autoclave expansion results of different cement−sand ratios.

Combination	Expansion Ratio (%)	Sample Description	Combination	Expansion Ratio (%)	Sample Description
#40 CS0.35 30−0	−−−−	Burst	CS0.25 30−0	−−−−	Burst
#40 CS0.35 30−30	0.080	Invariant	CS0.25 30−30	0.054	Invariant
#40 CS0.35 30−40	0.063	Invariant	CS0.25 30−40	0.049	Invariant
#40 CS0.35 50−0	−−−−	Burst	CS0.25 50−0	−−−−	Burst
#40 CS0.35 50−30	0.087	Invariant	CS0.25 50−30	0.056	Invariant
#40 CS0.35 50−40	0.061	Invariant	CS0.25 50−40	0.036	Invariant

**Table 7:** Characterization of fly−ash.

Elemental Composition	Al_2_O_3_	BaO	CaO	CO_3_O_4_	Cr_2_O_3_	CuO	Fe_2_O_3_	K_2_O	MgO	MnO	Na_2_O	NiO	P_2_O_5_	SO_3_	SiO_2_	TiO_2_
contents (%)	30.37	0.16	4.20	0.02%	0.02	0.01	5.24	1.01	1.20	0.05	0.65	0.02	1.00	1.16	53.61	1.29

**Table 8:** Characterization of EAF oxidizing slag.

Elemental Composition	SiO_2_	Al_2_O_3_	Fe_2_O_3_	CaO	MgO	K_2_O	SO_3_	TiO_2_	P_2_O_5_	MnO_2_
contents (%)	37.76	3.00	1.45	44.77	10.20	−−	−−	0.17	0.59	2.06

**Table 9:** Toxicity characteristic leaching procedure of the EAF oxidizing slag panel.

Elemental Composition Unit: ppm(mg/L)	As	Ba	Cd	Cr	Cr^6+^	Cu	Pb	Se	Hg
#4 CS0.35 30−40	0.004	0.643	ND	0.014	ND	ND	0.002	ND	ND
#4 CS0.35 50−40	0.007	0.680	ND	0.015	ND	ND	0.001	ND	ND
#40 CS0.35 30−40	0.005	0.569	ND	0.013	ND	ND	0.002	ND	ND
#40 CS0.35 50−40	0.005	0.628	ND	0.010	ND	ND	0.002	ND	ND
#40 CS0.25 30−30	ND	0.349	ND	0.083	ND	ND	0.009	ND	ND
#40 CS0.25 30−40	ND	0.343	ND	0.035	ND	ND	0.007	ND	ND
#40 CS0.25 50−30	ND	0.410	ND	0.046	ND	ND	0.008	ND	ND
#40 CS0.25 50−40	ND	0.415	ND	0.032	ND	ND	0.006	ND	ND
standard (ppm)	5.00	100.00	1.00	5.00	2.5	15.00	5.00	1.00	0.20

**Table 10:** Sound transmission losses of different combinations.

Combination	Frequencies (Hz)—Sound Transmission Losses (dB)													
	80	100	125	160	200	250	315	400	500	630	800	1000	1250	1600
#40 CS0.35 100−0	37.4	36.6	36.2	36.6	37.2	38.0	39.2	40.2	41.2	42.4	40.7	37.9	42.2	43.7
#40 CS0.35 30−30	44.7	43.1	41.9	43.4	44.5	45.9	47.4	47.5	49.6	52.3	51.6	51.7	51.4	49.3
#40 CS0.35 30−40	47.7	42.3	43.0	45.2	43.7	47.3	44.8	47.3	50.6	49.5	53.7	51.6	49.4	51.7
#40 CS0.35 50−30	41.5	40.6	41.6	39.4	40.7	43.5	44.7	48.1	50.9	54.0	52.1	52.2	48.7	46.9
#40 CS0.35 50−40	40.5	41.6	41.2	40.8	40.6	45.0	43.7	46.4	49.8	50.8	51.2	50.5	47.4	46.0
#40 CS0.25 30−30	39.6	39.9	39.5	38.7	39.1	43.2	43.0	44.3	47.0	50.9	49.9	47.1	48.7	47.0
#40 CS0.25 30−40	41.3	39.9	40.7	39.2	41.2	43.1	42.5	45.3	46.0	49.0	48.2	50.7	49.2	46.9
#40 CS0.25 50−30	40.9	39.5	40.3	38.1	39.1	42.8	41.2	43.4	45.0	48.5	47.8	49.3	49.4	46.1
#40 CS0.25 50−40	38.8	39.9	39.1	39.6	40.1	41.1	41.8	44.4	44.5	47.7	49.4	49.5	48.8	47.6

## Data Availability

The data presented in this study are openly available at “https://wsts.nstc.gov.tw/STSWeb/Award/AwardMultiQuery.aspx” (accessed on 1 August 2022).
